# Sulfated Chitosan Oligosaccharides Suppress LPS-Induced NO Production via JNK and NF-κB Inactivation

**DOI:** 10.3390/molecules191118232

**Published:** 2014-11-07

**Authors:** Jung-Hyun Kim, Yon-Suk Kim, Jin-Woo Hwang, Young-Ki Han, Jung-Suck Lee, Se-Kwon Kim, You-Jin Jeon, Sang-Ho Moon, Byong-Tae Jeon, Young Yil Bahk, Pyo-Jam Park

**Affiliations:** 1 Department of Biotechnology, Konkuk University, Chungju 380-701, Korea; 2 Industry-Academic Cooperation Foundation, Jeju National University, Jeju 690-756, Korea; 3 Specialized Graduate School & Technology Convergence, Department of Marine-Bio Convergence Science, Pukyong National University, Busan 608-737, Korea; 4 School of Marine Biomedical Sciences, Jeju National University, Jeju 690-756, Korea; 5 Korea Nokyong Research Center, Konkuk University, Chungju 380-701, Korea

**Keywords:** sulfated chitosan oligosaccharides, RAW264.7 cells, MAPKs pathways, anti-inflammatory

## Abstract

Various biological effects have been reported for sulfated chitosan oligosaccharides, but the molecular mechanisms of action of their anti-inflammatory effects are still unknown. This study aimed to evaluate the anti-inflammatory effects of sulfated chitosan oligosaccharides and to elucidate the possible mechanisms of action. The results showed that pretreated low molecular weight sulfated chitosan oligosaccharides inhibited the production of nitric oxide (NO) and inflammatory cytokines such as IL-6 and TNF-α in lipopolysaccharide (LPS)-activated RAW264.7 cells. The sulfated chitosan oligosaccharides also suppressed inducible nitric oxide synthase (iNOS), phosphorylation of JNK and translocation of p65, a subunit of NF-κB, into the nucleus by inhibiting degradation of IκB-α. Our investigation suggests sulfated chitosan oligosaccharides inhibit IL-6/TNF-α in LPS-induced macrophages, regulated by mitogen-activated protein kinases (MAPKs) pathways dependent on NF-κB activation.

## 1. Introduction

**Figure 1:**
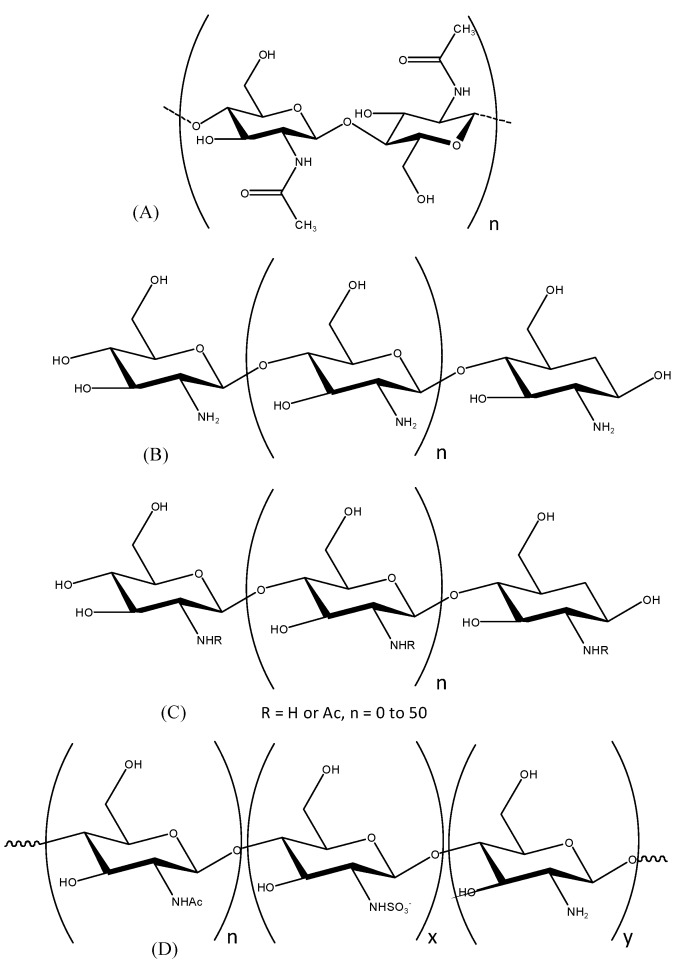
Chemical structure of (**A**) chitin; (**B**) chitosan; (**C**) chitooligosaccahrides; (**D**) sulfated chitosan oligosaccharide.

Chitosan oligosaccharides (COSs) are partially hydrolyzed products of chitosan, a biopolymer composed of β-(1-4)-linked *N*-acetyl-D-glucosamine and deacetylated glucosamine units [1]. Chitosan oligosaccharides (Figure 1) have been studied for their various biological effects, including anti-oxidative, anti-tumor, anti-microbial, anti-inflammation, and anti-coagulant properties. In addition, these derivatives could show much improved antioxidant activity compared with chitosan because of the diminished effects of the hydrogen bonds [2,3,4,5].

Sulfated polysaccharides display a flexible number of sulfate groups at fixed positions on the molecular backbone, which gives them their characteristic functional properties [6]. However, there are no reports on the anti-inflammatory effects of sulfated chitosan oligosaccharides. Hence, this study was undertaken to assess the mechanisms of the anti-inflammatory activity of sulfated chitosan oligosaccharides (S-COSs) *in vitro*.

Macrophages play important roles in immune responses against various microbial and viral infections by the secretion of nitric oxide (NO) and pro-inflammatory cytokines such as tumor necrosis factor-α (TNF-α), and interleukin-6 (IL-6) [7,8]. NO, a short-lived free radical, mediates many biological functions such as host defense, neurotransmission, neurotoxicity and vasodilation [9,10]. NO is synthesized endogenously by nitric oxide synthases (NOSs) through the conversion of L-arginine to NO and L-citrulline [11,12]. Three type of NOS isoforms were identified; neuronal NOS (nNOS), endothelial NOS (eNOS) and inducible NOS (iNOS). The latter is abundantly expressed and released upon activation by LPS in macrophages and thus simulates different inflammatory diseases including tissue injury and septic shock [13,14]. Toll-like receptor 4 (TLR4) is the extra-cellular receptor that recognizes LPS and causes the release of inflammatory mediators via two basic pathways, namely the myeloid differentiation factor-88 (MyD88) pathway and the TIR domain-containing adaptor inducing interferon-β (TRIF) pathway.

The MyD88-dependent pathway mediates activation of mitogen-activated protein kinases (MAPKs) such as extracellular signal-related kinase, p38 or c-Jun NH2-terminal kinase (JNK) and PI3K/Akt [15]. These kinases regulate expression of inflammatory genes via activation of downstream NF-κB by degrading IκB-α [16]. iNOS expression is essential for the activation of MyD88-dependent and TRIF-dependent signaling pathways in LPS-stimulated macrophages [17].

Based on all this information, in the present study we aimed to investigated the anti-inflammatory potential of sulfated chitosan oligosaccharides (S-COSs) and its molecular mechanism of action, the involvement of the MAPKs signaling pathway was studied, and the role played by nuclear factor-κB (NF-κB) was elucidated in LPS-stimulated RAW264.7 macrophage cells.

## 2. Results

### 2.1. Preparation of Chitooligosaccharides (COSs) and Sulfated COSs (S-COSs)

Three kinds of COSs, identified as HMWCOSs, MMWCOSs and LMWCOSs, were successfully prepared from 90% deacetylated chitosan using an UF membrane reactor system. To improve the biological activity, sulfated chitosan oligosaccharides were prepared according to our previous method [18]. In general, trimethylamine-sulfur trioxide is known to affect selective *N*-sulfation of amino alcohols [19], and it supplements the use of the pyridine-SO_3_ complex and related reagents with chitosan reported earlier [20,21]. In this study, sulfated COSs such as S-HMWCOSs, S-MMWCOSs and S-LMWCOSs were obtained in over 90% yields from HMWCOSs, MMWCOSs and LMWCOSs as white, fluffy and water-soluble materials. This synthetic mechanism was substitution by sulfate at the C-2, C-3, and C-6 positions [22]. Characteristic absorptions derived from the sulfo groups in the IR spectrum at 800, 1240, and 1350 cm^−1^ were assigned to C-O-S, S=O, and S-N, respectively [18].

### 2.2. Effects of Sulfated Chitosan Oligosaccharide (S-COS) Pretreatment on Cell Viability of LPS-Induced Macrophages

**Figure 2:**
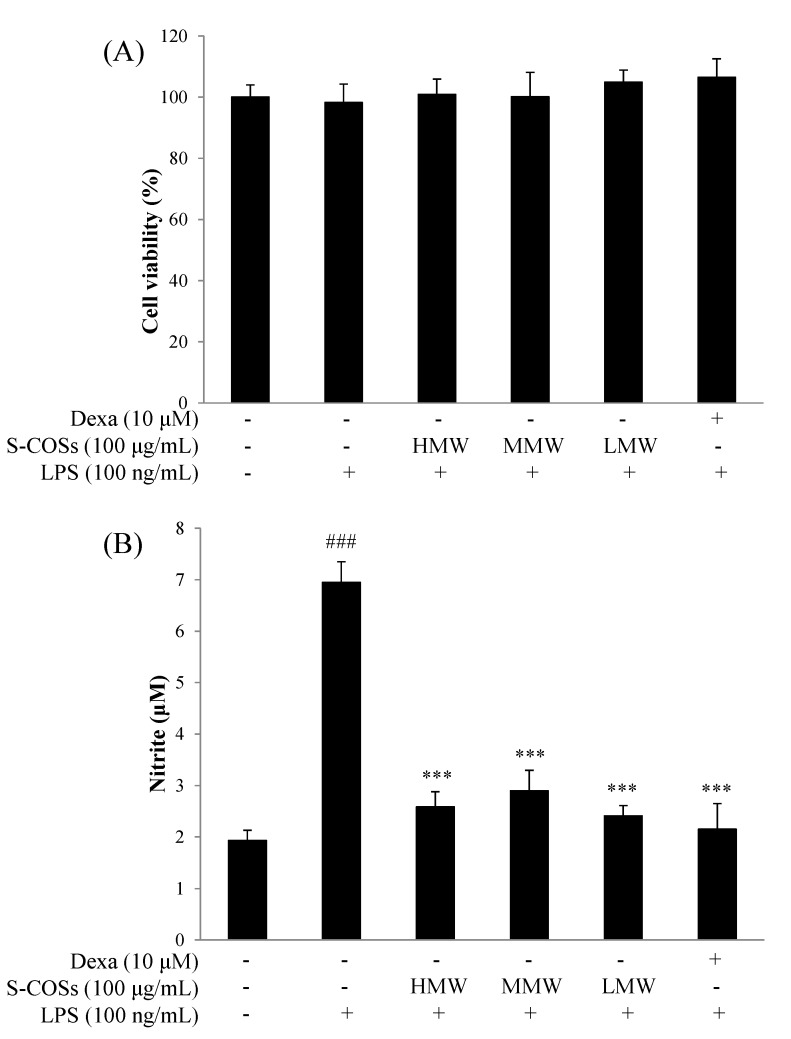
Cell viability (**A**) and nitrite production (**B**) of sulfated chitosan oligosaccharides (S-HMWCOSs, S-MMWCOSs and S-LMWCOSs) by LPS-stimulated RAW264.7 cells. RAW264.7 cells were treated with sulfated chitosan oligosaccharides (S-HMWCOSs, S-MMWCOSs and S-LMWCOSs) at 100 μg/mL for 24 h. Cell viability was examined by MTT assays. The concentration of NO in the culture medium was determined by using the Griess assay. Results are expressed as mean ± SEM from three independent experiments. ^###^ *p* < 0.001 *versus* control, *** *p* < 0.001 *versus* LPS alone group. Dexamethasone (Dexa) used as positive control.

The cytotoxicity of sulfated chitosan oligosaccharides to RAW264.7 cells was measured by MTT assays. RAW264.7 macrophage cells were treated with sulfated chitosan oligosaccharides (S-HMWCOSs, S-MMWCOSs, S-LMWCOSs) at 100 μg/mL for 24 h. As shown in Figure 2A, sulfated chitosan oligosaccharides are not cytotoxic in RAW264.7 cells at concentrations up to 100 μg/mL.

### 2.3. Effects of Sulfated Chitosan Oligosaccharides (S-COSs) on LPS-Induced Production of NO in Macrophages

**Figure 3:**
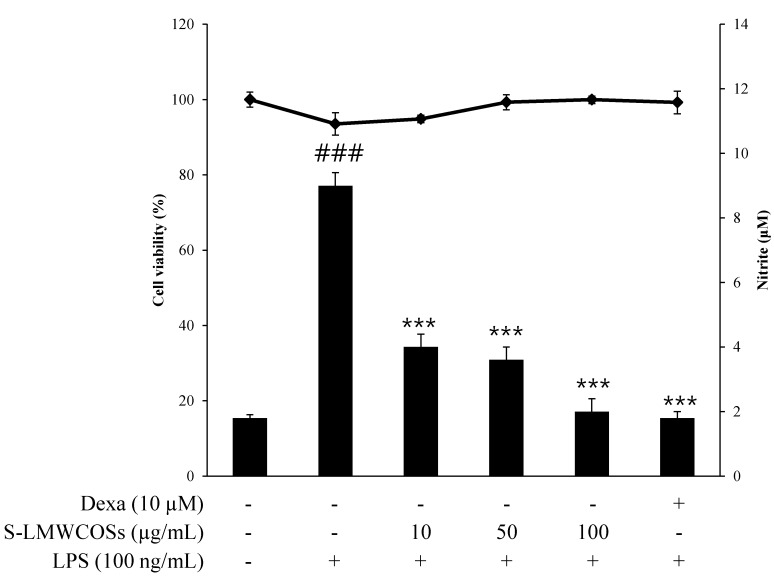
Cell viability and nitrite production of sulfated low molecular chitosan oligosaccharides (S-LMWCOSs) in LPS-stimulated RAW264.7 cells. RAW264.7 cells were treated with S-LMWCOSs (10, 50 and 100 μg/mL) in the absence of LPS for 24 h. Cell viability was examined by MTT assays. The concentration of NO in the culture medium was determined by using the Griess assay. Results are expressed as mean ± SEM from three independent experiments. ^###^ *p* < 0.001 *versus* control, *** *p* < 0.001 *versus* LPS alone group. Dexamethasone (Dexa) used as positive control.

To investigate whether sulfated chitosan oligosaccharides regulate NO production, RAW264.7 macrophages were incubated with 100 ng/mL LPS and sulfated chitosan oligosaccharides (S-COSs) for 20 h. In unstimulated RAW 264.7 cells, NO production were almost undetectable, but LPS- stimulated cells significantly increased the accumulation of nitrite in the culture medium by approximately 4-fold, and this increase was inhibited by sulfated chitosan oligosaccharides (S-COSs) (Figure 2B). As shown in Figure 2B, the pretreated low molecular weight sulfated chitosan oligosaccharides prepared from 90% deacetylated chitosan (S-LMWCOSs) showed the most potent NO production reduction in LPS-induced RAW264.7 cells. We then tested the anti-inflammation effects of the S-LMWCOSs with various concentrations (10, 50 and 100 μg/mL) and S-LMWCOSs significantly reduced the level of NO production in LPS-induced RAW264.7 cells in a dose-dependent manner (Figure 3).

### 2.4. Inhibitory Effects of S-LMWCOSs Pretreatment on LPS-Induced ROS in Macrophages

**Figure 4:**
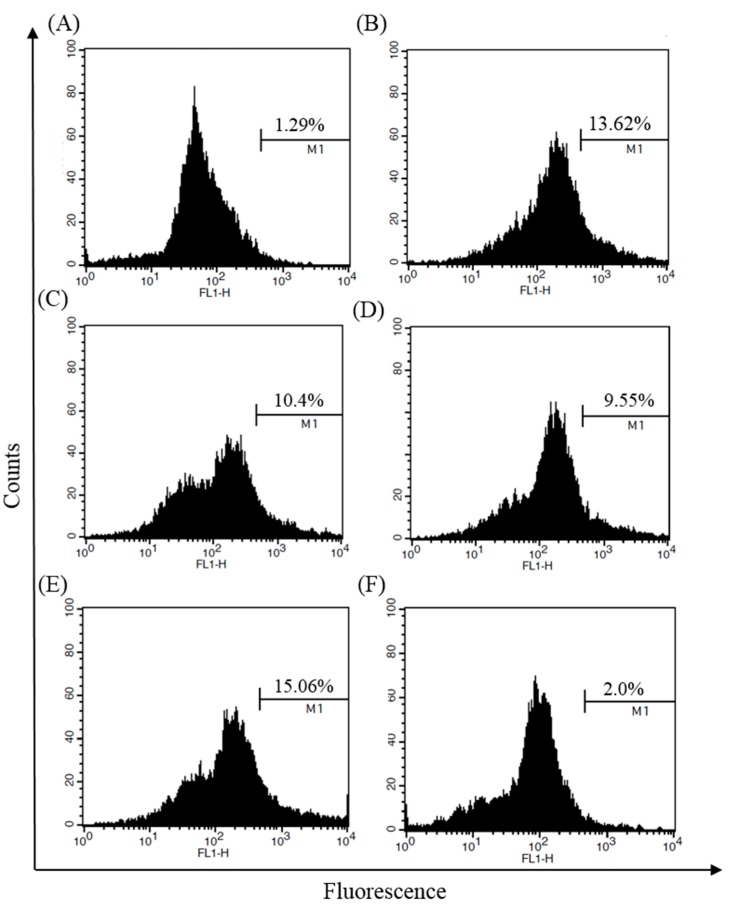
Inhibitory effects of S-LMWCOSs pretreatment on LPS-induced ROS in macrophages. RAW264.7 cells were incubated with control (**A**), LPS (**B**), S-LMWCOSs 10 μg/mL (**C**), S-LMWCOSs 50 μg/mL (**D**), S-LMWCOSs 100 μg/mL (**E**) and Dexamethasone 10 μM (**F**) for 24 h and quantitation of ROS was analyzed by FACS. The results shown are from one representative experiment repeated twice, with similar results.

Reactive oxygen species (ROS), which are synthesized by NADPH oxidase, serve as secondary messengers to activate multiple intracellular proteins and enzymes involved in physiological and pathological states. During inflammation, activated macrophages greatly increase the oxygen uptake resulting in a massive release of ROS called respiratory burst [23]. Overproduction of ROS is thought to be harmful in inflammatory diseases, so we examined the inhibition of ROS production by S-LMWCOSs in LPS-stimulated macrophages. As shown in Figure 4, intracellular ROS production in RAW264.7 cells due to LPS-induced oxidative damage declined when treated with S-LMWCOSs (10, 50 and 100 μg/mL).

### 2.5. Inhibitory Effects of S-LMWCOS Pretreatment on LPS-Induced Pro-Inflammatory Cytokines in Macrophages

**Figure 5:**
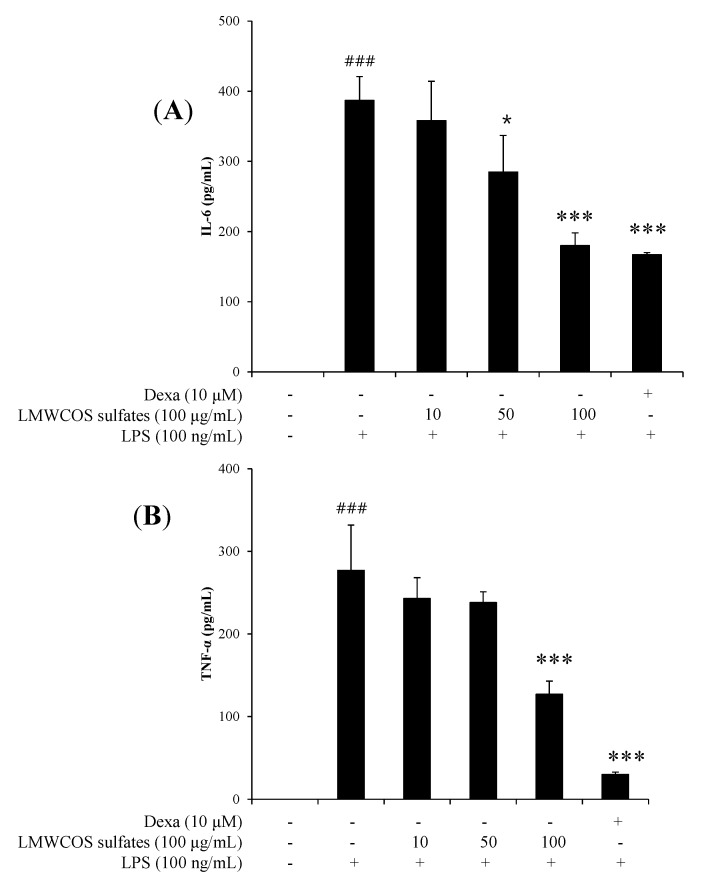
The effects of LMWCOS sulfates on pro-inflammatory cytokines by LPS-stimulated RAW264.7 cells. RAW264.7 cells were treated with the indicated doses of S-LMWCOSs 1 h before LPS treatment (100 ng/mL). After incubation of 24 h, the supernatants were taken, and the amounts of IL-6 (**A**) TNF-α (**B**) were measured by ELISA. Dexamethasone (Dexa) used as positive control. Results are expressed as mean ± SEM from three independent experiments. ^###^ *p* < 0.001 *versus* control, * *p* < 0.05, *** *p* < 0.001 *versus* LPS alone group.

To investigate the effects of S-LMWCOSs on proinflammatory cytokine production, ELISA assays were used to measure TNF-α and IL-6 levels in culture media of RAW264.7 cells pretreated with or without S-LMWCOSs (10, 50 and 100 μg/mL) and dexamethasone as a positive control for 1 h and stimulated with LPS for 2 h. Stimulation of RAW264.7 cells with LPS increased the secreted levels of TNF-α, and IL-6, which were significantly reduced by S-LMWCOSs in a dose-dependent manner (Figure 5A,B). The IC_50_ value of TNF-α, and IL-6 is 93.58 μg/mL, 94.82 μg/mL. In this study, we clearly confirmed that S-LMWCOSs dramatically reduced TNF-α and IL-6 in LPS-induced RAW264.7 cells compared to those of LPS-treated only RAW264.7 cells.

### 2.6. Inhibitory Effects of S-LMWCOSs Pretreatment on LPS-Induced iNOS and COX-2 in Macrophages

**Figure 6:**
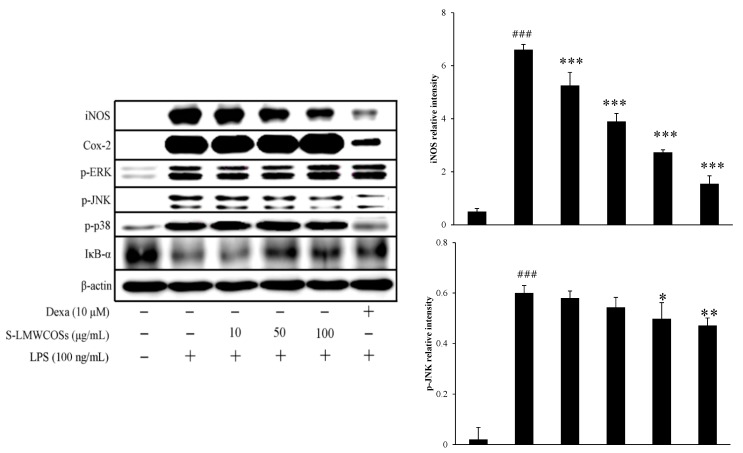
Effects of S-LMWCOSs on the regulation of various protein expression levels in LPS-stimulated RAW264.7 cells. iNOS, Cox-2, p-ERK, p-JNK, p-p38, IκB-α and actin expression levels were determined by western blotting. Dexamethasone (Dexa) used as positive control. The results shown are from one representative experiment repeated twice, with similar results. ^###^ *p* < 0.001 *versus* control, * *p* < 0.05, ** *p* < 0.01, *** *p* < 0.001 *versus* LPS alone group.

In order to elucidate the mechanism we investigated the effect of S-LMWCOSs on iNOS and COX-2 protein levels. We observed that various concentrations (10, 50 and 100 μg/mL) of S-LMWCOSs inhibited iNOS expression in the LPS-stimulated RAW264.7 cells (Figure 6). We found that iNOS protein was strongly expressed after stimulation with LPS, and the pre-treatment of S-LMWCOSs markedly decreased the level of iNOS protein in a concentration-dependent manner. As compared to LPS (100%) the decrease in iNOS for 10 μg/mL was 42% (60% NO release), 59% (78% NO release) for 50 μg/mL and 74% (80% NO release) for 100 μg/mL. However we did not observe any significant effect of S-LMWCOSs in LPS-induced COX-2 protein levels.

### 2.7. Inhibitory Effects of S-LMWCOS Pretreatment on LPS-Induced Phosphorylation of MAP Kinase and Activation of NF-κB in Macrophages

The MyD88-dependent pathway plays a critical role in the regulation of macrophage activation by activation of mitogen-activated protein kinases (MAPKs). MAPKs including ERK, JNK and p38 also play essential roles in regulation of pro-inflammatory cytokine production. It is known that LPS-induced phosphorylation of MAPKs and IκB-α lead to expression of pro-inflammatory mediators in macrophages [24]. To investigate whether the MAPKs pathway was regulated by S-LMWCOSs, we examined the phosphorylation of three MAPKs (ERK, JNK and p38). As shown in Figure 6, S-LMWCOSs decreased LPS-induced phosphorylation of JNK, but didn’t affect phosphorylation of p38 and ERK. As a positive control, dexamethasone suppressed the LPS-induced JNK and p38 MAP kinase phosphorylation, without ERK phosphorylation.

**Figure 7:**
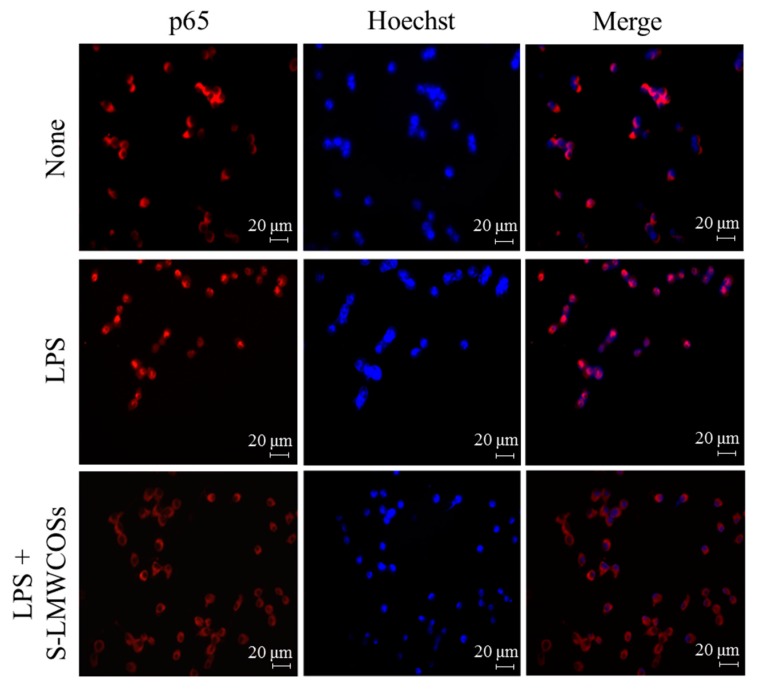
Immunofluorescence staining. The RAW264.7 cells were seeded at the density of 5 × 10^4^ cells/well on the 24-well plate. Cells were stimulated with LPS (100 ng/mL) in the absence or presence of S-LMWCOSs (100 μg/mL) added 1 h before the stimulation. At 30 min after the LPS addition, sub-cellular location of the NF-κB p65 subunit was determined by immunofluorescence assay. Cells were treated with the indicated dose of S-LMWCOSs 30 min before LPS (100 ng/mL) treatment. The stained cells were visualized using a fluorescence microscope at 200× magnification.

NF-κB is an important transcription factor in mediating the proinflammatory responses. In unstimulated cells, NF-κB is located in the cytoplasm as an inactive complex bound with IκB-α. After the activation by phosphorylation or ubiquitination, NF-κB disassociated with IκB-α and translocate into nucleus to initiate the transcription of target genes [25]. Therefore, we investigated the degradation of IκB-α by western blotting to describe the influence of S-LMWCOSs on LPS-induced NF-kB activation which is consistent with the influence on MAPKs signaling (Figure 6). We observed that phosphorylation of IkB by LMWCOSs (100 μg/mL) was similar to that shown by dexamethasone. Immunofluorescence staining showed that LPS stimulation caused noticeable translocation of NF-κB p65 from the cytoplasm into the nucleus, which was obviously counteracted by S-LMWCOSs pretreatment (Figure 7).

## 3. Discussion

In this study, we examined the potential anti-inflammatory effects of sulfated chitosan oligosaccharides in a LPS-stimulated inflammatory model using RAW264.7 cells and investigated the underlying signaling pathway. Macrophage-derived NO plays important roles in physiology, pathology and inflammatory responses [26]. However, overproduction of NO leads to a variety of diseases such as atherosclerosis, malignancy, rheumatoid arthritis, tissue injuries and septic shock [27,28], therefore, down-regulation of NO is very important for the treatment of these diseases. Before conducting experiments we also confirmed that the selected doses of S-LMWCOSs (10, 50 and 100 μM) did not induce cellular toxicity. To explore the mechanism of inhibition of NO production in RAW264.7 macrophages, the effect of sulfated chitosan oligosaccharides on iNOS protein expression was examined. Sulfated low molecular weight chitosan oligosaccharides (S-LMWCOSs) inhibited the expression of iNOS protein in dose-dependent manner.

During the inflammation process, macrophages actively participate in inflammatory responses by releasing the pro-inflammatory cytokines tumor necrosis factor-α (TNF-α) and IL-6, as well as other inflammatory factors, such as NO and prostaglandins (PGs), that recruit additional immune cells to the sites of infection or tissue injury [29]. In this study, we found that S-LMWCOSs dramatically reduced TNF-α and IL-6 in LPS-induced RAW264.7 cells compared to those of LPS-treated only RAW264.7 cells. LPS is known to activate mainly two different signal pathways, the MyD88- dependent (MAPKs and PI3K/Akt) and TRIF-dependent (IFN-β/STAT) pathways [30].

The MAPK pathway is one of the important intracellular signaling which cascades in pro-inflammatory responses. MAPKs comprise three subfamilies: p38 MAPK, JNK/stress activated protein kinase and ERK [31,32]. In this study, we found S-LMWCOSs inhibited LPS-induced iNOS in RAW 264.7 macrophages via JNK, but not p38 MAPK and ERK1/2. Many reports of the differential regulation of MAPKs on LPS-induced NO signaling has been described. Intracellular reactive oxygen species (ROS) have been implicated in the activation of NF-kB in various cell types including macrophages [33]. Recent studies have demonstrated that various natural compounds including ligustilide, resveratrol and curcumin revokes the accumulation of intracellular ROS, suppresses NF-kB activation and inhibits iNOS expression [34,35]. Ligustilide suppressed the LPS-induced production of NO, PGE2 and TNF-α by inhibiting both IKK/NF-κB and MAPK/AP-1 signaling, these effects may be related to the inhibition of LPS-induced intracellular ROS [36].

NF-κB is a major factor regulating LPS-induced inflammatory mediators, including iNOS, COX-2, TNF-α and IL-6 by blocking IκB-α degradation in the cytosol and the nuclear translocation of the NF-κB p65 subunit [37]. Therefore, the suitable regulation of NF-κB may be beneficial in treating many inflammatory disorders.

## 4. Experimental Section

### 4.1. Chemicals and Reagents

90% Deacetylated chitosan prepared from crab shells was donated by Kitto Life Co. (Pyeongtaek, Korea). The chitosanase (35,000 U/g protein) from *Bacillus* sp. was purchased from Amicosen Co. (Jinju, Korea), and an ultrafiltration (UF) membrane reactor system (Minitan^TM^) for the production of hetero-COSs was from Millipore Co. (Bedford, MA, USA). Dulbecco’s modified eagle’s medium (DMEM), fetal bovine serum (FBS), penicillin and streptomycin were purchased from Hyclone (Logan, UT, USA). Cell culture wares were purchased from BD Falcon (Franklin lakes, NJ, USA). Antibodies for β-actin, iNOS and cyclooxygenase (COX)-2 were purchased from Santa Cruz Biotechnology Inc. (Dallas, TEX, USA). Antibodies for phosphorylated-ERK, -p38 and -JNK were purchased from Cell Signaling Technology Inc. (Denver, MA, USA). The detection agents and polyvinylidine fluoride (PVDF) membrane were purchased from Amersham Biosciences (Piscataway, NJ, USA). Lipopolysaccharide (LPS), 2',7-dichlorofluorescein diacetate (DCFH-DA) and 3-(4,5-dimethylthiazol-2-yl)-2,5-diphenyltetrazolium bromide (MTT) were purchased from Sigma Chemical Co. (St. Louis, MO, USA). All other reagents were of the highest grade available commercially.

### 4.2. Preparation of Chitooligosaccharides (COSs)

COSs, which are COSs prepared from 90% deacetylated chitosan, were prepared by hydrolysis of chitosan in an UF membrane reactor system according to our previous method [18]. Briefly, a 1% solution of chitosan was prepared by dissolving chitosan (100 g) in distilled water (1.0 L) and 1.0 M lactic acid (550 mL), and the volume was made up to 10.0 L with distilled water. The pH was adjusted to 5.5 with a saturated sodium carbonate solution. An UF membrane reactor system was used for the fractionation of COSs. 90% Deacetylated chitosan was hydrolyzed with an endo-type chitosanase (35,000 U/g protein) with a substrate-to-enzyme ratio of 1:1.5 units for 36 h in a batch reactor, and was then heated at 98 °C for 10 min to inactivate the enzyme. Thereafter, the hydrolysates were separated using an UF membrane reactor system. The UF membranes used in the system were molecular weight cutoffs (MWCO) 10, 5, and 1 kDa, respectively. Chitosan oligosaccharides (COSs) were fractionated into three kinds of COSs with relatively high molecular weights (5000–10,000 Da; HMWCOSs), medium molecular weights (1000–5000 Da; MMWCOSs), and low molecular weights (below 1000 Da; LMWCOSs). For detailed information mentioning the molecular weight of COSs please refer the work by Park *et al*. [18].

### 4.3. Preparation of Sulfated Chitooligosaccharides (S-COSs)

Sulfated chitooligosaccharides (S-COSs) were prepared according to our previous method [18]. COSs (10 g) which had previously been lyophilized were dispersed in distilled water (1.0 L), and was treated with 2.2 g sodium carbonate anhydrous and 4.5 g trimethylamine-sulfur trioxide. The mixture was heated at 65 °C until a clear viscous solution was obtained for about 12 h. The cooled mixture was then dialyzed exhaustively against distilled water using an electronic dialyzer (Micro Acilyzer G3, Asahi Chemical Industry Co., Tokyo, Japan), and lyophilized. The dialyzer membrane used was Aciplex Cartridge (AC-230–400). Freeze-dried dialyzed samples were obtained as light brown fluffy powders, which were dissolved in distilled water, filtered through 0.2 M filter and used for *in vitro* experiments. We prepared three kinds of sulfated derivatives (S-HMWCOSs, S-MMWCOSs, S-LMWCOSs) from three kinds of COSs.

### 4.4. Cell Culture

Murine macrophage RAW264.7 cells from the American Type Culture Collection (ATCC, TIB-71^TM^) were cultured in DMEM medium supplemented with antibiotics (100 units/mL of penicillin and 100 μg/mL of streptomycin) and 10% heat-inactivated FBS and maintained at 37 °C in a humidified incubator containing 5% CO_2_.

### 4.5. Determination of Cell Viability Assay

RAW264.7 cells were plated at a density of 1 × 10^4^ cells/well into 24-well plates, pre-incubated for 1 h with three kinds of sulfated chitosan oligosaccharides (S-COSs) at 100 μg/mL, various concentrations (10, 50 and 100 μg/mL) of low molecular weight sulfated chitosan oligosaccharides (S-LMWCOSs) and dexamethasone (10 μM, as a positive control), and stimulated with or without 100 ng/mL of LPS in medium at 37 °C for 24 h. The cells were subsequently incubated in 500 μL of MTT solution (0.2 mg/mL) at 37 °C for 4 h. The formazan formed was dissolved in DMSO and measured at a wavelength of 540 nm using a microplate reader.

### 4.6. Determination of Nitric Oxide (NO) Production

NO concentrations in cultured medium were measured as nitrite by the Griess reagent [38]. RAW264.7 cells were plated at a density of 1 × 10^4^ cells/well into 24-well plates, pre-incubated with three kinds of sulfated chitosan oligosaccharides (S-COSs), various concentrations (10, 50 and 100 μg/mL) of S-LMWCOSs and dexamethasone (10 μM, as positive control), and stimulated with 100 ng/mL of LPS in medium at 37 °C for 24 h. Each cultured medium (100 μL, supernatant) was mixed with 100 μL of Griess reagent and incubated at room temperature for 10 min. The absorbance of the mixture was determined at 540 nm using a microplate reader. All measurements were performed in triplicate. The nitrite levels were determined through a standard curve established with NaNO_2_.

### 4.7. Reactive Oxygen Species (ROS) Assay

The level of intracellular peroxides was determined by labeling with cell-permeable, 7-dichlorofluorescin diacetate (DCFH-DA) as described previously [39]. Briefly, DCFH-DA (10 μM) was added to pre-incubated sulfate chitosan oligosaccharides in RAW264.7 macrophages for 30 min at 37 °C in dark, and cells were gently scraped. Fluorescence intensity was analyzed at an excitation wavelength of 485 nm and an emission wavelength of 535 nm using a FACS Calibur flow cytometer (Becton & Dickinson Co., Franklin lakes, NJ, USA).

### 4.8. Measurement of Cytokine (IL-6 and TNF-α)

RAW264.7 cells were plated at a density of 1 × 10^4^ cells/well in 24-well plates, pre-treated with various concentrations (10, 50 and 100 μg/mL) of S-LMWCOSs and dexamethasone (10 μM) for 1 h, and stimulated with 100 ng/mL of LPS in medium at 37 °C for 2 h and 18 h. The levels of IL-6 and TNF-α in cultured medium (supernatant) were measured by enzyme-linked immunosorbent assay (ELISA) according to the manufacturer’s protocols. The absorbance was determined at 450 nm using a microplate reader.

### 4.9. Western Blot Analysis

RAW264.7 cells were plated at a density of 2 × 10^5^ cells in 6-cm dish, pre-treated with various concentrations (10, 50 and 100 μg/mL) of S-LMWCOSs and dexamethasone (10 μM) for 1 h, and stimulated with LPS (100 ng/mL). The cells were subsequently washed with PBS, collected, suspended in the lysis buffer (150 mM NaCl, 10 mM Tris (pH 7.5), 5 mM EDTA, 1% Triton X-100) containing protease inhibitors (1 μg/mL leupeptin and 100 μg/mL PMSF) and centrifuged at 12,000 g at 4 °C for 20 min to yield cell lysates. The protein level in each sample was measured using a protein assay kit (Bio-Rad, Laboratories, Inc., Hercules, CA, USA). The proteins (20 μg of RAW264.7 lysates) were separated with 10% SDS polyacrylamide gels and transferred to polyvinylidene difluoride (PVDF) membranes (GE Healthcare, Little Chalfont, Buckinghamshire, UK). The membranes were then blocked in Tris-buffered saline (TBS)-Tween 20 solution containing 5% non-fat dry milk and incubated sequentially with primary antibody and horseradish peroxidase-conjugated anti-goat or anti-rabbit IgG (Bio-Rad). The protein bands were then visualized using an ECL detection kit by Luminescent image analyzer (LAS-3000, Fujifilm, Tokyo, Japan).

### 4.10. Immunofluorescence Assay

To determine the intracellular location of the p65 subunit of NF-*κ*B, RAW264.7 cells (1 × 10^4^ cells/well in 24-well plate) were cultured on sterile cover slips in 24-well plates and pretreated with different concentrations of S-LMWCOSs and dexamethasone (10 μM) for 1 h. At 30 min after the LPS treatment, the cells were fixed with methanol for 20 min at −20 °C and washed with PBS for 5 min. The fixed cells were permeabilized with 1% Triton X-100 in PBS for 1 h at room temperature, washed three times with PBS for 5 min. The permeabilized cells were then treated with 1 mg/mL of monoclonal mouse antihuman NF-κB (p65) for 60 min at room temperature and then washed with 0.05% Tween-20 in PBS for 5 min. Cells were then incubated in a 1:2000 dilution of Alexa Fluor 568—labeled goat anti-mouse antibody for 60 min at room temperature, washed with PBS for 5 min. Cells were then stained with 0.1 μM of Hoechst staining solution for 20 min at RT and then washed. Finally, the cover slips with cells were dried at RT in an oven for 45 min and mounted in a 1:1 mixture of xylene and malinol. More than 50 cells per field were counted under a fluorescence microscope.

### 4.11. Statistical Analysis

The data are expressed as mean standard deviation for triplicate determinations. Analysis of variance (ANOVA), accompanied with Tukey’s test and Dunnett’s test (GraphPad Prism 5), were conducted to identify the significant differences between samples (*p* < 0.05).

## 5. Conclusions

Our findings indicate that low molecular sulfated chitosan oligosaccharides suppressed the pro-inflammatory mediators such as NO and iNOS in LPS-stimulated murine macrophage RAW264.7 cells. The probable molecular mechanisms behind this bioactivity might be due to inhibition of LPS-induced IL-6 and TNF-α release, by down-regulating the phosphorylation levels of MAPK signaling pathways, decreasing IκB-α degradation and subsequent NF-κB activation in RAW264.7 cells. Although the existing data demonstrated an effect of S-LMWCOSs in a cellular model, it would be interesting to reproduce the effect in an animal model of inflammation. Together this data might confirm an anti-inflammatory activity of S-LMWCOSs that might serve as a novel therapeutic for disorders wherein inflammation is one of the pathological features.
